# Infrared nanospectroscopic imaging of DNA molecules on mica surface

**DOI:** 10.1038/s41598-022-23637-4

**Published:** 2022-11-08

**Authors:** Irma Custovic, Nicolas Pocholle, Eric Bourillot, Eric Lesniewska, Olivier Piétrement

**Affiliations:** Laboratoire Interdisciplinaire Carnot de Bourgogne (ICB), UMR CNRS 6303, Université de Bourgogne-Franche-Comté, 9 Avenue Alain Savary, 21078 Dijon Cedex, France

**Keywords:** Biophysics, DNA and RNA, Imaging techniques, Scanning probe microscopy

## Abstract

Significant efforts have been done in last two decades to develop nanoscale spectroscopy techniques owning to their great potential for single-molecule structural detection and in addition, to resolve open questions in heterogeneous biological systems, such as protein–DNA complexes. Applying IR-AFM technique has become a powerful leverage for obtaining simultaneous absorption spectra with a nanoscale spatial resolution for studied proteins, however the AFM-IR investigation of DNA molecules on surface, as a benchmark for a nucleoprotein complexes nanocharacterization, has remained elusive. Herein, we demonstrate methodological approach for acquisition of AFM-IR mapping modalities with corresponding absorption spectra based on two different DNA deposition protocols on spermidine and Ni^2+^ pretreated mica surface. The nanoscale IR absorbance of distinctly formed DNA morphologies on mica are demonstrated through series of AFM-IR absorption maps with corresponding IR spectrum. Our results thus demonstrate the sensitivity of AFM-IR nanospectroscopy for a nucleic acid research with an open potential to be employed in further investigation of nucleoprotein complexes.

## Introduction

The assessment of structure-depended function and interactions of biomolecules has been greatly hinged on the development of diverse spectroscopic techniques^1–4^. In that context, a particularly applicable approach is vibrational spectroscopy, owning to its leverage to retrieve bond-specific chemical information assigned to molecular characterization and its correlative structural properties^5–9^. The most essential cellular biological processes depend on complex interplay between nucleic acids and proteins and a quantitative assessment of these interactions is crucial for understanding mechanisms which govern deoxyribonucleic acid (DNA) replication, transcription, recombination or DNA repair^10^. While the latter are increasingly well-characterized by conventional vibrational spectroscopy on ensemble level^11–15^, however understanding the nanoscopic nucleoprotein-level interactions, or lack thereof, remains a prominent challenge. In general, low-dimensional biological phenomena occur at nanometer scale size, whereas the applications of conventional vibrational spectroscopy are constrained due to low spatial-resolution performance set by optical diffraction limit, which could at its best approach 300 nm depending on the excitation source. Since bulk spectroscopy could be challenging to apply for biological heterogeneous systems, such us DNA–protein complexes due to acquisition of the chemical information averaged on the ensemble level of molecular spices, the behaviour of individuals and their internalization mechanism has been addressed by single-molecule spectroscopy which could provide a profound impact on how we understand bimolecular processes^4,16^.

A widely adapted label-based technique for single-molecule detection in biochemical research is fluorescence spectroscopy providing a temporal fluorescence-encoded resolution of biomolecules under studies^17,18^. In particular, the fluorescence spectroscopy has been effectively used for investigation of distinct structural behaviour with an access to molecular observable dynamic resulting from protein–DNA interactions^19,20^. Along with label-based assessment, the understanding of interactions among biomolecules could be contingent on the label-free spatial imaging modalities, with high sensitivity at nanoscale area over the nanometre length scale of relevance to biological systems.

Apropos of various chemical nanoimaging techniques, two nanoscale analogues Raman and Infrared spectroscopy coupled with atomic force microscopy, as tip-enhanced Raman spectroscopy (TERS) and atomic force microscopy-based infrared spectroscopy (AFM-IR), facilitate a common platform enabling nanometer spatially resolved chemical spectroscopy without substantial sacrifice of sensitivity and spatial resolution^21–26^. Both techniques became of particular interest for biological systems, as has been reported recently to demonstrate nanoscale (< 20 nm) vibrational insight in constitutes of bacteria^27^, individual vesicles^28^, oligomer and fibrillar aggregates during amyloid formations^29^, protein aggregates^29–32^, protein-based process^33,34^, towards descending size cellular constructs such as methylation status in single human metaphase chromosome^35^ as well secondary structure of single protein molecule^36^.

Since the advent of atomic force microscopy (AFM), deoxyribonucleic acid (DNA) is considered as a benchmark sample, against which new technical developments are tested^37,38^. In current state-of-the-art, TERS technique has provided tremendous sensitivity and applicability in nucleic acid research^22,26^, In that context, the TERS has been used for a sequencing procedure of single stranded DNA molecule and chemically identifying a single base-pair resolution^39^. As complemental nanospectroscopy method sharing the same scanning probe platform, yet based on different physical mechanism, AFM-IR advantageously combines the nanoscale spatial resolution provided by AFM together with the chemical information offered by IR spectroscopy^21,23^. Unlike conventional IR spectroscopy, AFM cantilever is used as detector to sense localized IR-absorption induced thermal expansion thus providing nanoscale resolution-related chemical information through IR spectrum^40^. The outstanding advantage of AFM-IR technique is thus direct measuring IR absorption as a function of position across the sample, creating colorized AFM-IR chemical maps that correspond to distribution of chemical species localized at the nanoscale. Although recently, Knowls et al. have demonstrated first acquisition of infrared absorption spectra and chemical maps of protein at the single molecule level^36^, surprisingly to our knowledge, AFM-IR has not been used for nanocharacterization of a single DNA molecule, to date.

The recent improvement of AFM-IR sensitivity by integration of resonance enhanced cantilever oscillation with high repetition rate laser allows measurement of ultra-thin samples down to monolayer, thus providing an interest to be employed into DNA research, despite its small dimeter (about 2 nm) and demanding surface properties^41–43^. We propose here to reach this challenging goal by nanochemical mapping of DNA molecules deposited onto mica surface thus pushing the horizons further in applicability of AFM-IR technique for nucleic acid research. Intrinsically, the thermo-mechanical properties of sample and the dynamics of AFM cantilevers are crucial to acquire AFM-IR data, and as such, has a great potential to simplify methods of nanoscale characterization of DNA molecules. In that context, DNA plasmids and mica surface, as underlying substrate, could be used as a standard sample to perform AFM-IR nanocharacterization. Given the reported knowledge of DNA deposition on surfaces for conventional AFM studies, one can state that atomically flat mica surface is preferable substrate that permits a broad range of DNA deposition methods while providing high signal-to-noise ratio (SNR) images^44^. Therefore, we present first reported methodological approach for acquisition of AFM-IR absorption mapping modality and corresponding spectrum of DNA on mica surface employing short-term deposition based on biochemical relevant protocols.

## Methods

Primarily, summarizing the reported knowledge, two main strategies are established to bind negatively charged DNA onto negatively charged mica surface: (1) polyelectrolyte-coated mica surface with polyamines-based chemical compounds such as spermidine, spermine, or poly-l-lysine^45,46^ and (2) through metal counterions mediated DNA adsorption^47,48^. From the first DNA adsorption strategy, we have chosen spermidine-pretreatment method which usually forms self-assembled monolayer with exposed positively charged amino groups NH^3+^ that results in attraction of DNA through counterions interaction^46^. The second chosen strategy for DNA binding upon mica surface is based on mica pretreatment by transition metal cations such as Ni^2+^, which simultaneously enhances the DNA fixation and reduces the repulsive contribution, proposed by model of electrical double-layer force^47^.

### Mica surface

**Figure 1:**
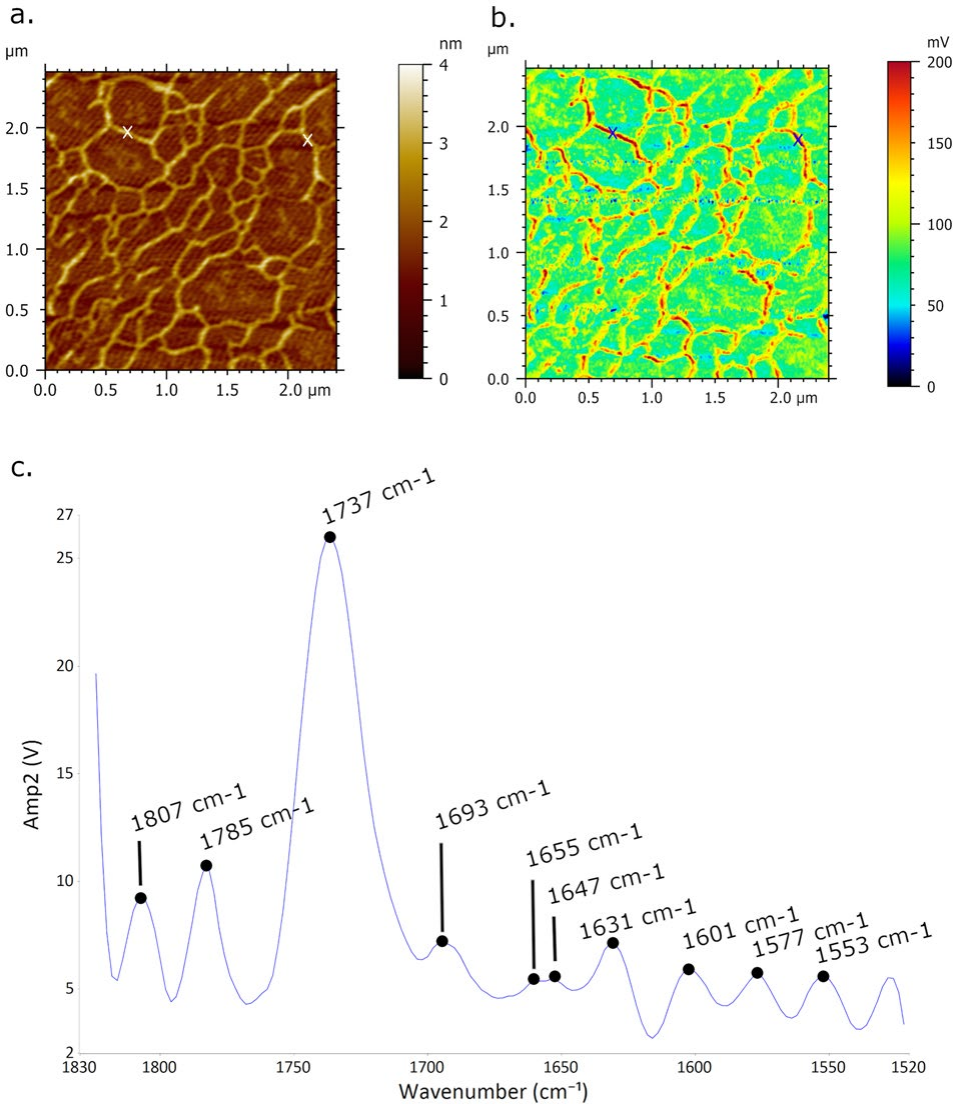
AFM-IR imaging of DNA network formed upon spermidine pretreated mica surface. Size of imaged area: 2.4 × 2.4 µm. (**a**) AFM topography image of DNA and corresponding. (**b**) AFM-IR absorption map of DNA network recorded at optimized wavenumber 1728 cm^−1^ showing DNA of higher absorbance (bright red porous contour) regarding to underlying mica surface. (**c**) AFM-IR spectra acquired and collected from two positions of DNA (blue cross in (**a**) and (**b**)) showing prominent broaden band peaks of C=O stretching with absorption maxima at 1737 cm^−1^.

**Figure 2:**
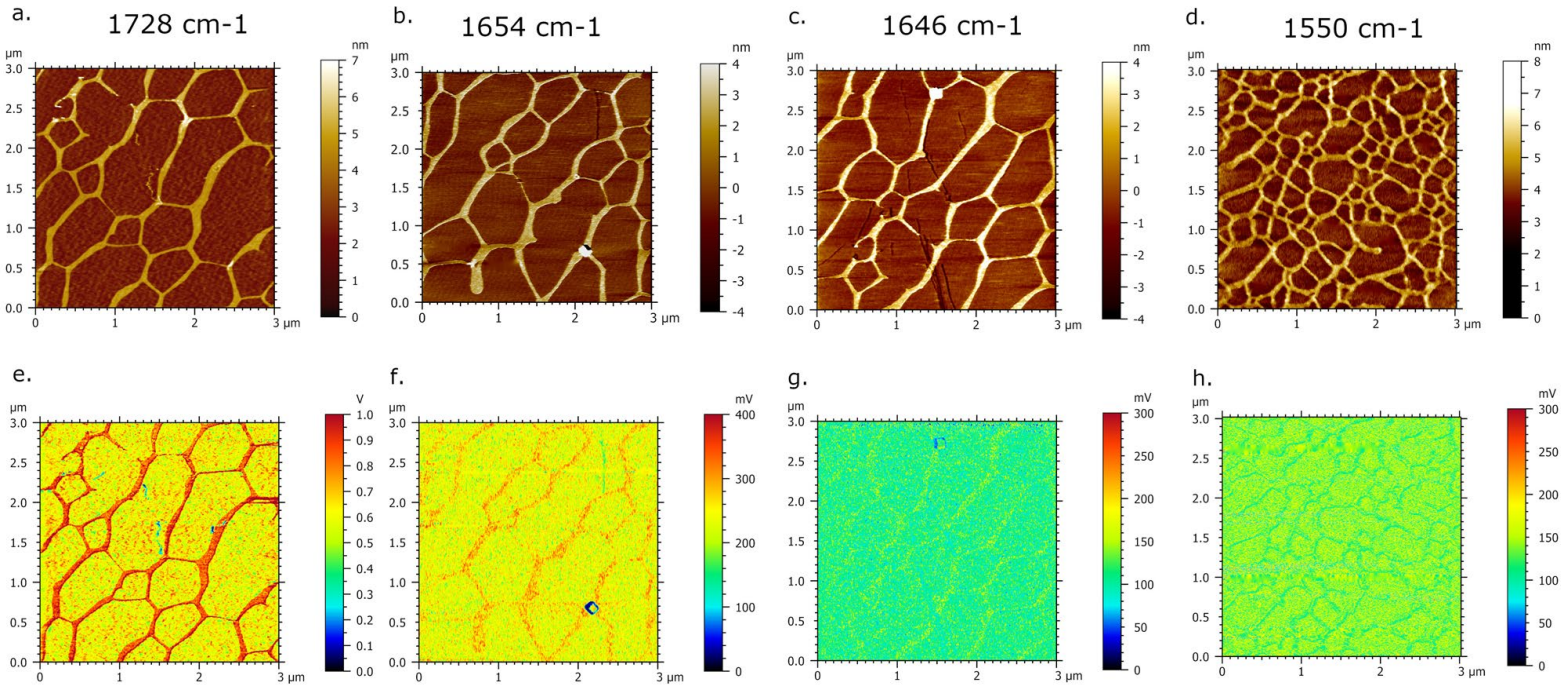
AFM-IR imaging of DNA network deposited onto spermidine-functionalized mica surface. (**a**–**d**) AFM topography images and corresponding. (**e**–**h**) IR absorption maps of DNA network with a highest contrast-absorption of DNA acquired at 1728 cm^−1^, gradually attenuated at 1654 and 1646 cm^−1^ and highest contrast-absorption of background at 1550 cm^−1^.

Following the deposition onto mica surface, AFM-IR characterization of DNA requires specific conditions to be conductive including DNA installation upon AFM-IR prism. Prior to installation upon CaF_2_ prism, the DNA-mica sample was cleaved on the samples’ backside by scotch tape in order to obtained as transparent-possible mica surface for efficient IR laser light transmissions (see Fig. S1). The effect of the mica surface thickness on IR amplitude is presented in Fig. 1b (non-optimized thickness of the mica surface) and Fig. 2e (optimized thickness of the mica surface). Both of the figures present the AFM-IR absorption maps of DNA networks, scanned at the same frequency 1728 cm^−1^. The amplitude of IR absorption is lower in Fig. 1b (200 mV) regarding to the Fig. 2e (1 V) because the thickness of mica in that stage of the experiment was not thin (or transparent) enough to enhance IR light transmission (as it was the case for the experiment displayed in Fig. 2e). It is important to note that thickness of mica installed upon prism effects the amplitude but not the contrast of DNA on recorded AFM-IR absorption map. Based on qualitative evidence, yet reproducible one, the amplitude of DNA absorption on AFM-IR absorption map is higher once the scanning is acquired upon mica surface with reduced thickness (see Fig. S1). After, the DNA sample is transferred upon CaF_2_ pyramid prism (source Crystran) and fixed with type N immersion liquid (Leica Microsystems).

### Preparation of DNA samples

We purchased puC19 DNA plasmid (New England Biolabs), and we linearized it with EcoRI enzyme (New England Biolabs). After 60 min of incubation, we rinsed the solution with Tris–HCl 10 mM (pH 7.5), NaCl 1 M buffer solution through Amicon Ultra-0.5 30 kDa (Millipore). DNA is finally eluted to 10 µg/mL in TE buffer [10 mM Tris–HCl (pH 7.45), 1 mM EDTA] and aliquots of 50 µL were frozen and stored at − 20 °C.

### Deposition of DNA network upon spermidine pretreated mica surface

We diluted DNA in ionic buffer [10 mM Tris, 20 mM NaCl_2_ and 5 mM MgCl_2_] down to 5 µg/mL and flash frozen. We used Spermidine 0.1 M solution (Sigma-Aldrich Chemicals Co) stored at 4 °C and diluted at 50 µM. On freshly cleaved mica surface (Muscovite Mica Sheets 15 mm diameter disk, V1 quality, EMS), we added 5 µL of DNA ionic-buffer solution upon spermidine-pretreated mica area and incubated for 3 min. Each deposition step (spermidine and DNA-buffer solution) was followed by drying mica surface with gentle touch of filter paper.

### Deposition of single molecules DNA on Ni^2+^ pretreated mica surface

We diluted DNA in ionic-deposition buffer [10 mM Tris–HCl (pH = 7.45), 10 mM MgCl_2_, 25 mM KCl] down to 5 µg/mL and stored at 4 °C overnight. The protocol of single molecule DNA deposition is adapted from Perkins et al.^49^ We pretreated mica surface with a 20 μL drop of 100 mM NiCl_2_ (Sigma-Aldrich) onto the freshly cleaved mica for 1 min followed by rinsing with 50 mL of ultrapure water.

The mica was then quickly dried by touching filter paper and completely drying the surface. Immediately after drying mica surface, we deposited 20 μL of DNA in ionic-deposition buffer where the concentration of the DNA was 1.5 µg/mL. After 2 s, we gently rinsed the surface with ~ 1 mL of deposition buffer followed by additional 8 mL of dewetting-based rinsing in portions of 150 µL. Finally, the surface was gently rinsed with 2 mL of imaging buffer [10 mM Tris (pH 7.5), 10 mM NiCl_2_ + 25 mM KCl]. During deposition and rinsing, solutions were kept at room temperature (19 °C for the room containing our AFM). At all other times, the salt reagents were kept at 4 °C. Buffers were filtrated with filter paper of 0.2 µm porosity each day from concentrated stocks.

### FT-IR experiment

Prior to IR-AFM imaging, we employed FTIR spectroscopy, to examine chemical-vibrational respond of deliberately chosen DNA network sample, due to extended DNA morphology which could provide enhanced IR signal as an outcome. For the spectrum of DNA deposited on spermidine-pretreated mica surface, characteristic IR absorption peaks can be observed at 1728 cm^−1^, 1654 cm^−1^, 1646 cm^−1^ in domain between 1800 and 1500 cm^−1^. In this domain, the DNA peaks are principally corresponding to in-plane-double bond stretching vibrations of the bases which are characteristic of various base pairing schemes.

### Conventional AFM imaging and Nano-IR AFM spectroscopy measurements

Prior to nanospectroscopy imaging, we performed conventional AFM imaging of DNA samples by Bruker AFM multimode in Peak Force Tapping Mode (silicon-nitride ScanAsyst-Air cantilever of spring Constant 0.4 N/m).

We performed nanospectroscopy measurements by NanoIR1 system (Anasys Instruments Inc., USA) which operates in Resonance Enhanced AFM-IR in contact mode at a scan rate of 0.6 Hz with a narrow-window of a low setpoint values (− 2.5 V until 0.25 V) avoiding the destruction contact-mode influence of the tip upon sample. The AFM scanning was performed by Al and Au coated HQ: CSC38/Al BS and HQ: CSC38/Cr-Au probes (Micromasch) having a spring constant of 0.03–0.09 N/m with resolution of 512 × 512 pixels and 128 × co-averages at the rate of 0.6 Hz. AFM-IR images were acquired in resonance Enhanced PLL mode (Phase-Locked-Loop) were feedback loop traced the contact resonance frequency of cantilever. We set laser power at 15%. We choose a second cantilever oscillation for obtaining the cantilever “ringdown signal” with the frequency centre of 171 kHz as shown in Fig. S7d. A standard procedure for successful AFM-IR imaging requires the optimization of IR laser spot.

We used Optimized function for the fine-tuning to search the IR spot in order to obtain periodic waveform deflection signal. The IR laser Spot position was optimized for three different frequencies (1728 cm^−1^, 1654 cm^−1^ and 1648 cm^−1^) subsequently, which appeared as the absorption bands in the acquired FTIR spectrum for DNA network (see Fig. S4b). The optimized window for each frequency is presented in Fig. S7. The resulting images are the maps of the IR Peak signal at each x and y location. The Fig. S7a–c show expected clean and distinct spot-like signal centralized under the cross-hair. During the scanning, we recommend to optimize IR laser focus several times, because the location of IR-Peak signal could be altered. We set the frequency window at 50 kHz for DNA network and 25 kHz for the single DNA molecule. Each spectrum is collected at the targeted position with the spectral resolution of 2 cm^−1^ and 128 × co-averages within the range of 1522–1824 cm^−1^ and duty cycle at 5% or 7% which depends of the sample. Then it was normalized using the Anasys software (Analysis Studio) and successively, it was smoothed with a Savitzky–Golay filter (third order, five points). All the AFM topography images and AFM-IR maps are treated (flattened and de-noising option) by using MountainsSPIP 8 software.

The factor of environmental conditions was one of the crucial to obtain higher IR absorption of DNA regarding to a mica surface. We have noticed that in the non-controlled ambient conditions, the AFM-IR absorption map shows DNA with a lower IR absorption regarding to the underlaying mica substrate see Fig. S2a,b. In order to avoid this problem and taking in consideration that DNA molecules are attracting humidity, we propose to set temperature at 25 °C and below with humidity lower than 25%.

## Results and discussion

### Nanoscale chemical imaging of DNA network formed upon spermidine pretreated mica surface

Polyamines, such as spermine and spermidine, are small organic polycations involved in diverse DNA-based biological processes and show prominent ability to bind and condensate DNA and chromatin^45,50,51^. In addition, polyamines are essential to AFM spreading for AFM imaging in all condition, and especially at high salt concentration^52^. Typical AFM image of DNA deposited upon spermidine-functionalized mica surface is presented in Fig. S3. The porous-like extended DNA network suggests that DNA molecules are attracted to each other, yet not strong enough to generate fully spherical-condensed structure. As a first attempt towards AFM-IR analysis of DNA molecules, we have deliberately chosen DNA network formed upon spermidine pretreated mica surface, due to its extended DNA morphology which could provide enhanced collection of AFM-IR signal as an outcome. We have used second cantilever oscillation mode with the frequency centre between 169–171 kHz (see Fig. S7d) and frequency window of 50 kHz. The AFM-IR spectra are collected in the range between 1520 and 1820 cm^−1^ where peaks principally correspond to in-plane double-bond stretching vibrations characteristic for various nucleobases in DNA^53^. Figure 1 presents AFM-IR imaging of DNA network formed upon spermidine pretreated mica surface with the infrared laser focus optimized at 1728 cm^−1^. The prime evidence of IR absorbance is presented by AFM-IR map (Fig. 1b) where DNA network is screened as an intense red porous-contour indicating higher IR absorption regarding to underlying mica surface (colorized as green background in Fig. 1b). The spectra acquired upon DNA network (pointed out by blue x-mark in Fig. 1b) obtained the prominent absorption band in range between 1700–1770 cm^−1^, as presented in Fig. 1c. Particularly, this broad absorption band was found to consist of overlapped in-plane vibration of C=O, C=C and C=N groups for heterocyclic basis^54^. In addition, spectral feature with the peak at 1693 cm^−1^ arises from adenin^55^. The observed absorbance bands with peaks at 1647 cm^−1^ and 1655 cm^−1^ are signature to C_2_=O_2_ medium strength stretching of cytosine and of amide I band, respectively^13,56^. A low intensity peak was found near 1630 cm^−1^ associated to amines groups^57^. As observable, the absorption band with peak found at 1577 cm^−1^ is assigned to C=N vibrational stretching of adenine^58^. Additionally, we tuned the laser to a fixed wavelength, taken as referenced from FTIR spectrum (see Fig. S4), in order to evaluate the IR absorbance respond of DNA network. Figure 2 presents AFM-IR topography images (a–d) recorded simultaneously with corresponding (e–h) AFM-IR absorption maps based on intensity of the IR signal at selected wavenumber values, annotated above the bottom row of Fig. 2. Each set (AFM image and AFM-IR map) were recorded directly after previous image once the optimization of IR laser light is done.

The absorption AFM-IR maps recorded at 1728 cm^−1^, 1654 cm^−1^ and 1646 cm^−1^ present the nanoscale IR absorption of DNA highest at 1728 cm^−1^ regarding to underlying mica surface (Fig. 2e), while gradually attenuated at 1654 cm^−1^ and 1646 cm^−1^ (Fig. 2f,g). The lowest IR absorption contrast among the series of AFM-IR maps in Fig. 2 was observed at 1550 cm^−1^ where the background is mapped with higher IR absorption regarding to DNA network, possibly originating from the IR absorption response of mica surface^59^.

### Nanoscale chemical imaging of single molecule DNA on mica surface

The second strategy for binding DNA onto mica surface is based on Ni^2+^ preincubated mica and three-steps based protocol with narrow ionic conditions^49^. The AFM image of 2.3 kbps single DNA molecule is presented in Fig. S5. adopting mixed A-form and B-form conformation^60,61^. For AFM-IR nanocharacterization of single DNA molecule deposited onto mica surface, we have chosen second cantilever oscillation mode for optimizing the cantilever ringdown signal at its frequency centre of 169 kHz and window of 25 kHz, instead of 50 kHz utilized for AFM-IR nanocharacterization of DNA network.

As a first step towards acquisition of AFM-IR absorbance map and spectrum of single DNA, we optimized laser focus at 1728 cm^−1^ which correspond to the highest DNA network absorbance among presented in series of absorption maps (see Fig. 2e). The single DNA bright yellow contour acquired at 1728 cm^−1^ in Fig. S6b presents higher DNA absorbance with the respect to the undelaying mica surface. The acquired and averaged spectra upon DNA molecule obtained broaden spectral band in region between 1678 cm^−1^ and 1780 cm^−1^ with absorption maxima, primarily at 1729 cm^−1^, shifted 8 cm^−1^ downward comparing to the maximum absorption for DNA network, and in addition at 1748 cm^−1^. These bands, along with shoulder peaks at 1690 cm^−1^ and 1700 cm^−1^ present overlapping of frequencies assigned to the base carbonyl starching and ring breathing mode of nucleobases^58^. In addition, spectral feature is observed at 1633 cm^−1^ corresponding to vibrations of C6=O of guanine and C4=O of thymine^62^. Peak with prominent intensity found at 1609 is assigned to adenine vibrations in DNA^63^ while peak with smaller intensity at 1539 cm^−1^ is contributed to in-plane vibration of cytosine^53^.

**Figure 3:**
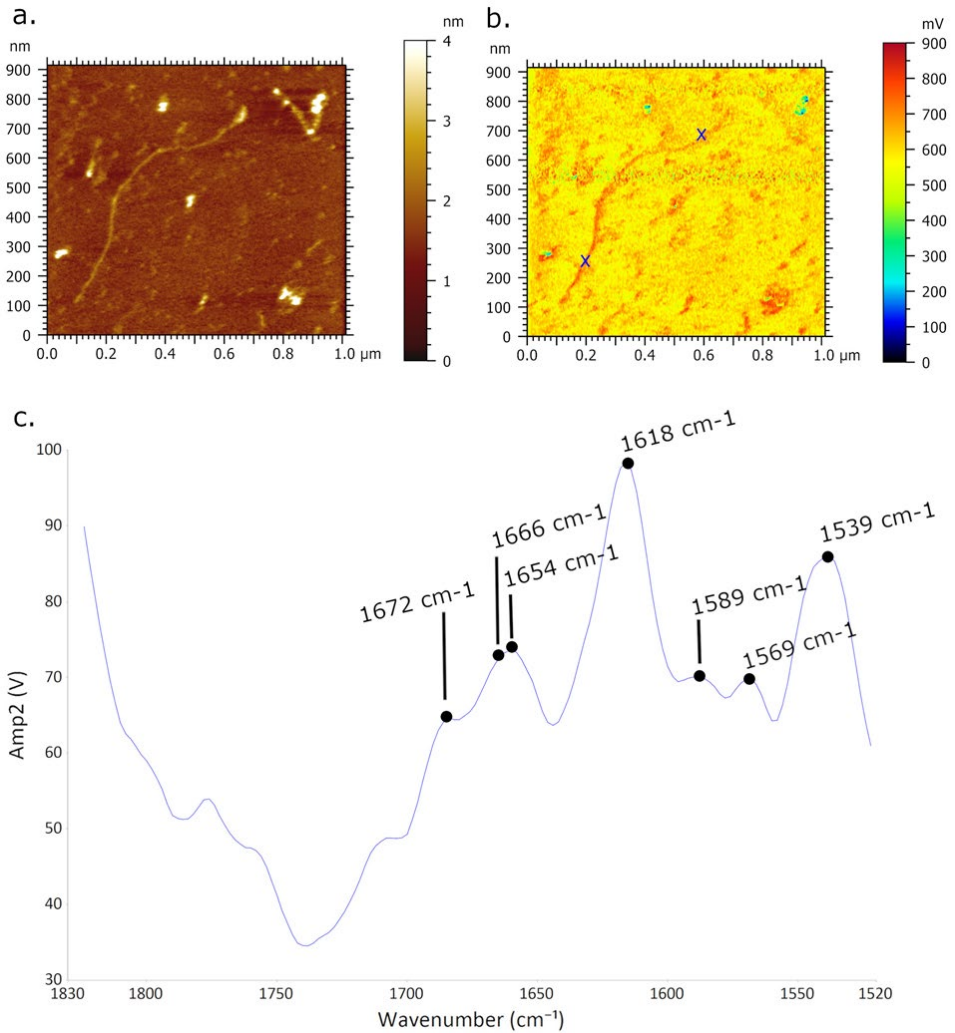
AFM-IR imaging of single DNA molecule deposited upon Ni^2+^ pretreated mica surface. Size of imaged area: 1.0 × 1.0 µm. (**a**) AFM topography image of DNA and corresponding. (**b**) AFM-IR absorption map of single DNA recorded at optimized wavenumber 1633 cm^−1^ showing DNA of higher absorbance (bright red contour) regarding to mica surface. (**c**) AFM-IR spectra acquired and collected from two positions of DNA (two blue crosses in (**b**)) showing prominent broadening peaks of amide bands with absorption maxima at 1618 cm^−1^.

**Figure 4:**
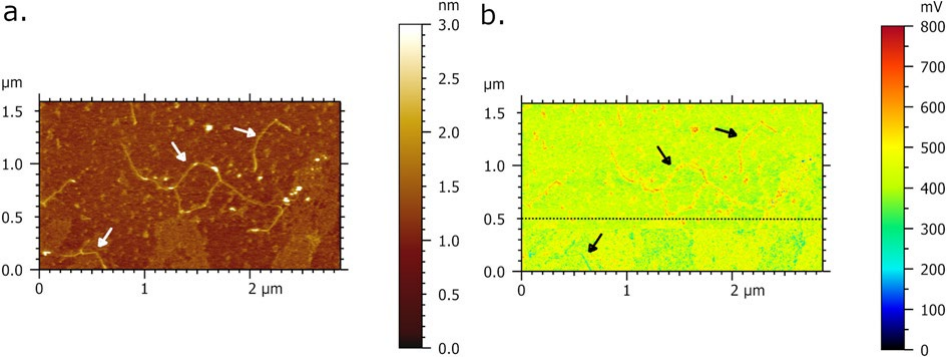
AFM-IR imaging of single DNA molecule deposited onto Ni^2+^-functionalized mica surface. (**a**) AFM morphology map and corresponding (**b**) AFM-IR absorption maps of single DNA with a higher (red contour) and lower (blue/green contour) absorbance acquired at laser pulse tune configuration with fixed frequencies at 1664 cm^−1^ (above dashed line) and 1550 cm^−1^ (below dashed line), respectively.

Figure 3 presents AFM-IR imaging of single DNA molecule on mica surface recorded with infrared laser focus optimized at 1633 cm^−1^. The higher IR absorbance is contributed to single DNA molecule regarding to underlying mica surface which is monitored by AFM-IR absorption map (Fig. 3b) as bright-red DNA contour. The acquired and averaged spectra (Fig. 3c) based on two positions on DNA molecule (pointed out by blue x-marks) obtained broadening of peaks of amide bands with absorption maxima at 1618 cm^−1^. Besides, the prominent spectral band has been found in region between 1520–1558 cm^−1^ with embedded peak at 1539 cm^−1^ assigned to Amide II band^58,61,64^. The prominent fingerprints of amide I band are found in the region between 1640–1670 cm^−1^ involving the carbonyl stretching modes C=O of nucleotide bases^6,53,58^. Within this band, two peaks are notable at 1654 cm^−1^ and 1664 cm^−1^, assigned to amide I and C2=O2 strength of cytosine, respectively, along with shoulder peaks^58^ at 1652 cm^−1^ and 1672 cm^−1^. A band with lower intensity is identified at 1568 cm^−1^ which corresponds to guanine C=N ring vibration^56^. Spectral feature observed at 1589 cm^−1^ is attributed to C–C phenyl ring stretches^58^. In order to monitor the sensitivity of IR detection based on single DNA molecule, Fig. 4 presents evident shifting of single DNA absorbance once the pulse laser is tuned at fixed frequencies 1664 cm^−1^ and 1550 cm^−1^. Thus, the single DNA shows the lower IR absorbance (part below the dashed line in Fig. 4b) acquired at 1550 cm^−1^, screened through the blue single DNA contour, which is in agreement with the lower DNA network absorption regarding to mica surface observed in Fig. 2h. In the same image, the higher DNA absorbance (part above the dashed line in Fig. 4b) is acquired at 1664 cm^−1^, and mapped as red DNA contour.

## Discussion

**Figure 5:**
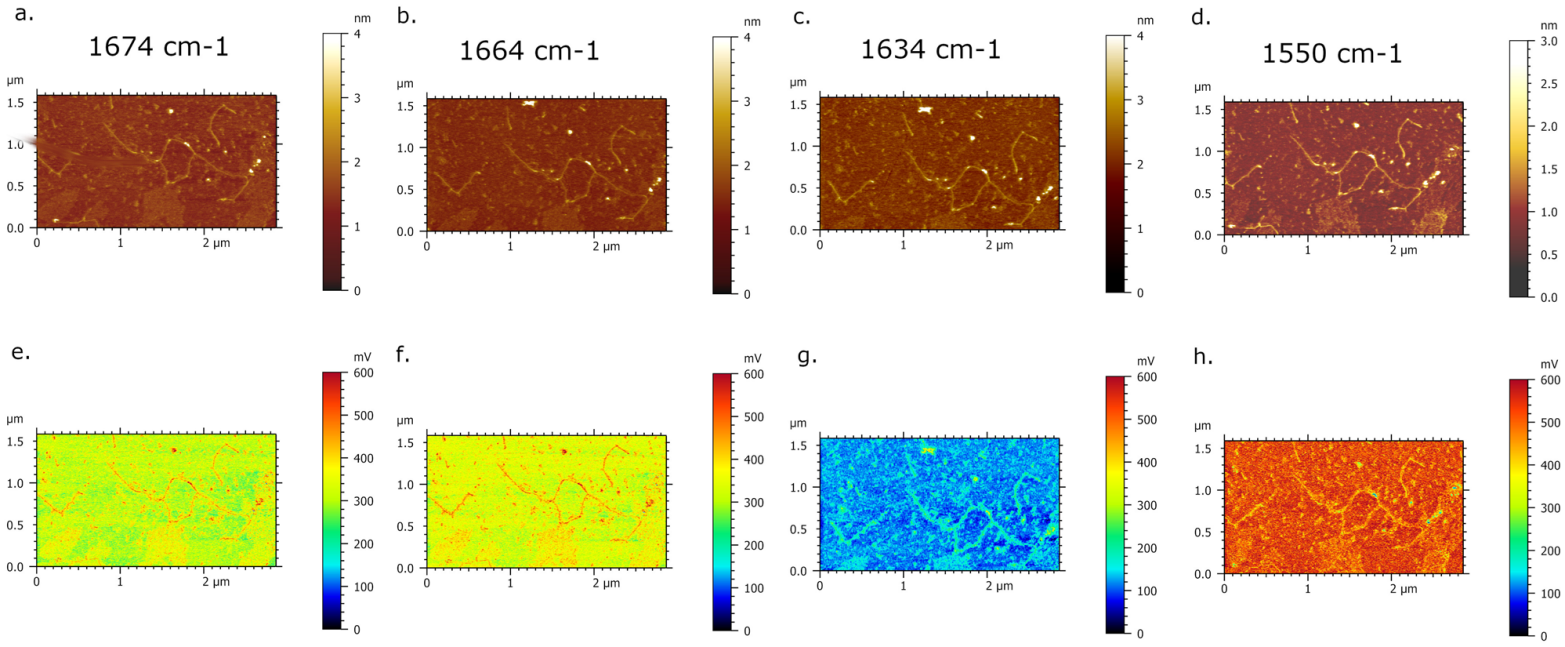
AFM-IR imaging of DNA single molecule deposited onto nickel functionalized mica surface. (**a**–**d**) AFM morphology map and corresponding (**e**–**h**) IR absorption maps of DNA single molecule with a highest contrast-absorption of DNA acquired at 1674 cm^−1^, 1664 cm^−1^, 1634 cm^−1^ and 1550 cm^−1^, respectively.

Prior to registration of AFM-IR imaging, we used Optimized function to search for IR spot (see Fig. S7 and “Methods”). We optimized the focused IR spot at three fixed wavenumbers corresponding to the major absorption. Hence, the resulting image of Optimized window show the highest spot-alike IR-peak signal at fixed wavenumber of 1728 cm^−1^. Consecutive registration of DNA network absorbance through AFM-IR maps at selected wavenumbers 1728 cm^−1^, 1654 cm^−1^ and 1648 cm^−1^ represent good agreement with obtained FT-IR data. In fact, the most intense absorbance of DNA network at AFM-IR maps is presented at 1728 cm^−1^ (outline by bright red porous contour in Figs. 1b and 2e). The discrepancy between the highest signal amplitude at FTIR spectrum (1648 cm^−1^) and the highest absorbance at AFM-IR maps (1728 cm^−1^) could possibly originate from different areas’ size of signal collection. In addition, we acquired multiple spectra upon DNA network at different wavenumbers (Fig. S9) and we constantly observed dominative absorption bands with the peak at 1737 cm^−1^ while the absence of a pretreated mica signal contribution to AFM-IR spectrum was also determined in Fig. S8. Indeed, the recent AFM-IR studies have reported the absorption map acquired at 1720 cm^−1^ dedicated to the guanine base which show spatially resolved absorbance of DNA-peptide complex^65^. In addition, Pancani et al., supported AFM-IR mapping of internal cells’ structures with confocal imaging and detected the absorption band between 1646 and 1760 cm^−1^ with the absorption shoulder at 1730 cm^−1^ assigned to the C=O starching of carbonyl groups and moreover, to the acid functions of nucleic acids in the nucleus^66^. The reported FTIR studies of DNA solution exhibited peaks at 1708 cm^−1^ and 1741 cm^−1^ which are associated to C=O stretching, particularly assigned to guanine C=O bonds^65^. Therefore, we could conclude that AFM-IR imaging of DNA network sample showed specific sensitivity for C=O stretching of carbonyl groups. The absence of a pretreated mica signal contribution to the reported AFM-IR spectra of DNA was also determined in Fig. S8. The influence of DNA sample preparation on AFM-IR spectrum have not been discussed to date. Generally, the polyamines at the low concentration bind strongly to DNA basis which is reflected through changes in intensity and shifting of the absorption band in region of in-plane DNA vibrational frequencies. In the context of the reported FTIR studies, the effected spectral region of spermidine–DNA interaction is observed at 1717 cm^−1^, 1663 cm^−1^ and 1600 cm^−1^, mainly due to the double bond in-plane stretching vibration of guanine (C7=N) located at the major groove and A–T base pair stretching, respectively. The prominent band at the AFM-IR spectrum (Fig. 1b), which appears in range between 1700 and 1770 cm^−1^, is likely embedding signal of C=O stretching, and in addition, possibly overlapping a signal of spermidine–DNA interaction^58,67,68^. As rendered evidence, each AFM-IR spectrums acquired at different wavenumbers upon DNA network (see Fig. S8a–c) show the matching intense signal within the range of 1700 cm^−1^ and 1770 cm^−1^. Thus, we can conclude that continual presence of this absorption band for formed DNA network is likely affected by DNA–spermidine interaction, along with the C=O stretching vibration of nucleobases. Otherwise, the AFM-IR spectrums acquired upon single DNA at different wavenumbers (see Fig. S9d–f) show peaks in the region preferably corresponding to amide I, amide II band and vibration fingerprints assigned to nucleobases. The AFM-IR spectrum acquired for single DNA at optimized wavenumber 1728 cm^−1^, show ambiguously the wider absorption band with the decreased amplitude and two maximum of the absorption, 1729 cm^−1^ and 1748 cm^−1^, distinctively to the AFM-IR spectrum of DNA network (see Fig. S9a,d). Since the polyamines may fulfil a role in gene regulation, we show that presented high sensitivity of AFM-IR technique could become the inception to for future study of polyamine-DNA induced structural effects at nanoscales. In addition, the acquired AFM-IR spectrum of single DNA molecule consists protruding contribution of nucleobases vibrational stretching. The consecutive registration of single DNA absorption through AFM-IR maps (see Fig. 5) at selected wavenumbers 1634 cm^−1^, 1664 cm^−1^ and 1674 cm^−1^ represent vibrations of C6=O of guanine and C4=O of thymine^62^, C2=O2 of cytosine^63^, C6=O of guanine and C4=O of thymine^69^. Therefore, based on acquired absorption maps and spectrum with a resolution of single DNA molecule, AFM-IR could become a powerful strategy for spectroscopic nanocharacterization and imaging of chemical-modified DNA and nucleoprotein complexes.

## Conclusion

We show in this work the first AFM-IR analysis of DNA, organized in network or on single molecule. The IR signal is highly sensitive to experimental conditions, such as temperature or humidity, but with right settings, we demonstrate that it is possible to chemically map DNA sample with a nanometer scale and with a high sensitivity. Indeed, to prepare DNA network we used pre-treated mica surface with spermidine, which binds also strongly to DNA, and we detected the presence of spermidine on IR spectrum. We believe that achievement of high AFM-IR sensitivity of deposited DNA molecules on mica surface are prerequisite for AFM-IR nanospectroscopic characterization of DNA–protein complexes in future work which could lead, for an instance, to the identification and the localisation of protein in multi-components assembly.

## Supplementary Information

Supplementary Figures.

## Data Availability

The datasets generated during and/or analyzed during the current study are available from the corresponding author on reasonable request.
