# Supplementary material for: Infrared nanospectroscopic imaging of DNA molecules on mica surface
Source: Sci Rep. 2022 Nov 8;12:18972. doi: 10.1038/s41598-022-23637-4 (PMC9643503; doi:10.1038/s41598-022-23637-4)
Supplement: Supplementary file 1 — Supplementary Figures. [file 41598_2022_23637_MOESM1_ESM.docx]

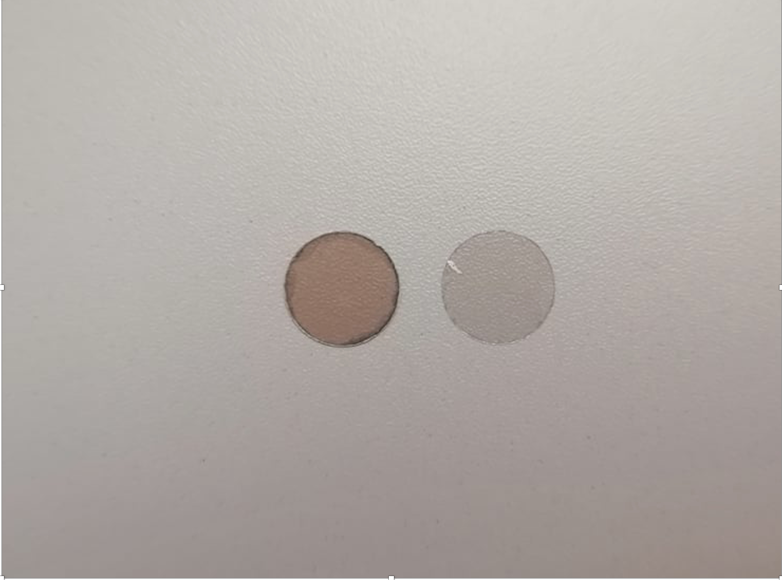


**Figure S1** Photography of muscovite mica substrate. Purchased mica substrate (Electron Microscopy Science) on left with thickness of 0.15-0.21 mm and scotch tape exfoliated mica substrate, on right, for AFM-IR experimental set-up.


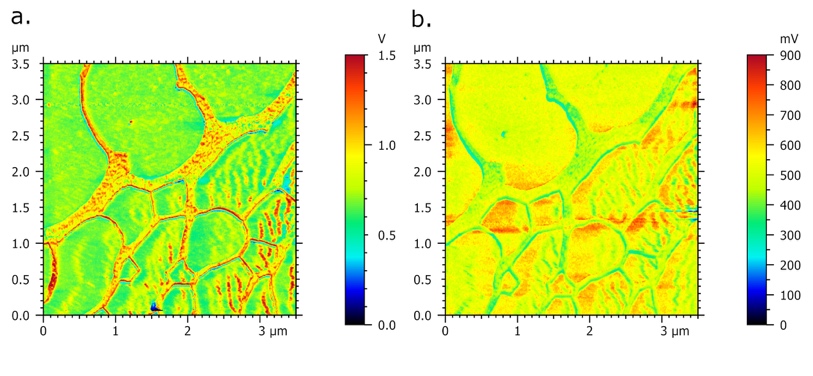


**Figure S.2** The effect of ambient humidity on AFM-IR absorption maps of DNA networked upon mica surface. a) IR-AFM absorbance map with higher IR absorbance of DNA (bright red contour) regarding to mica surface (underlying green colorization) mapped at 1728 cm^-1^. b) IR-AFM absorbance map with lower IR absorbance of DNA (green contour) regarding to mica surface (underlying yellow colorization) mapped at 1728 cm^-1^ at the ambient of humidity higher than 30 %.


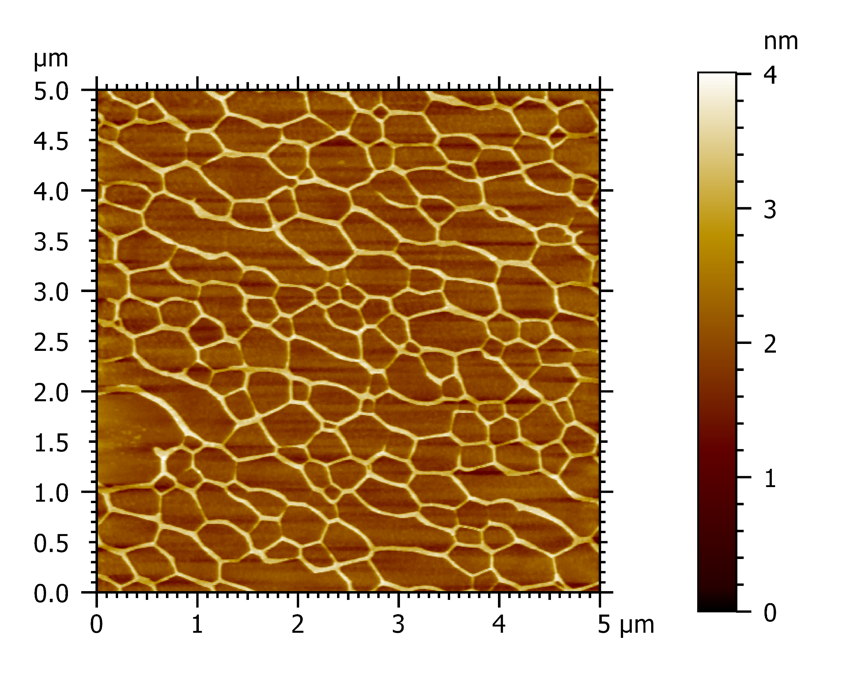


**Figure S3** AFM image of DNA formed network on spermidine pretreated-mica surface. AFM topography.


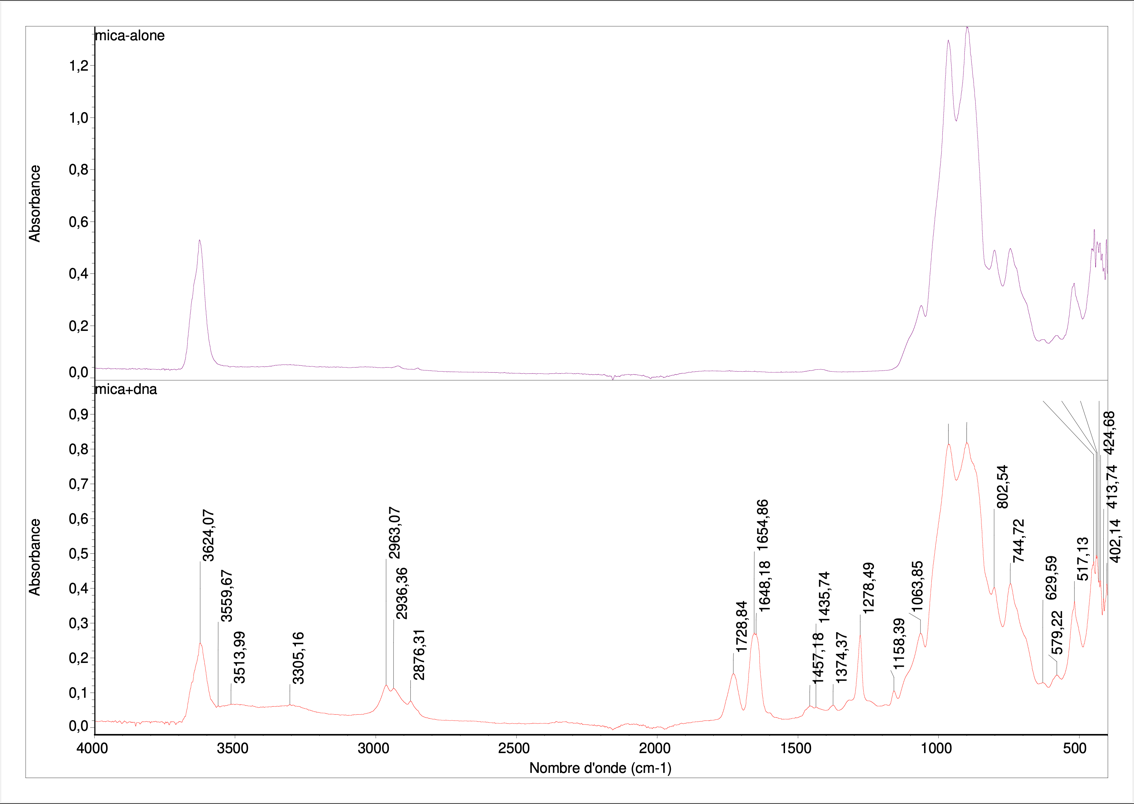

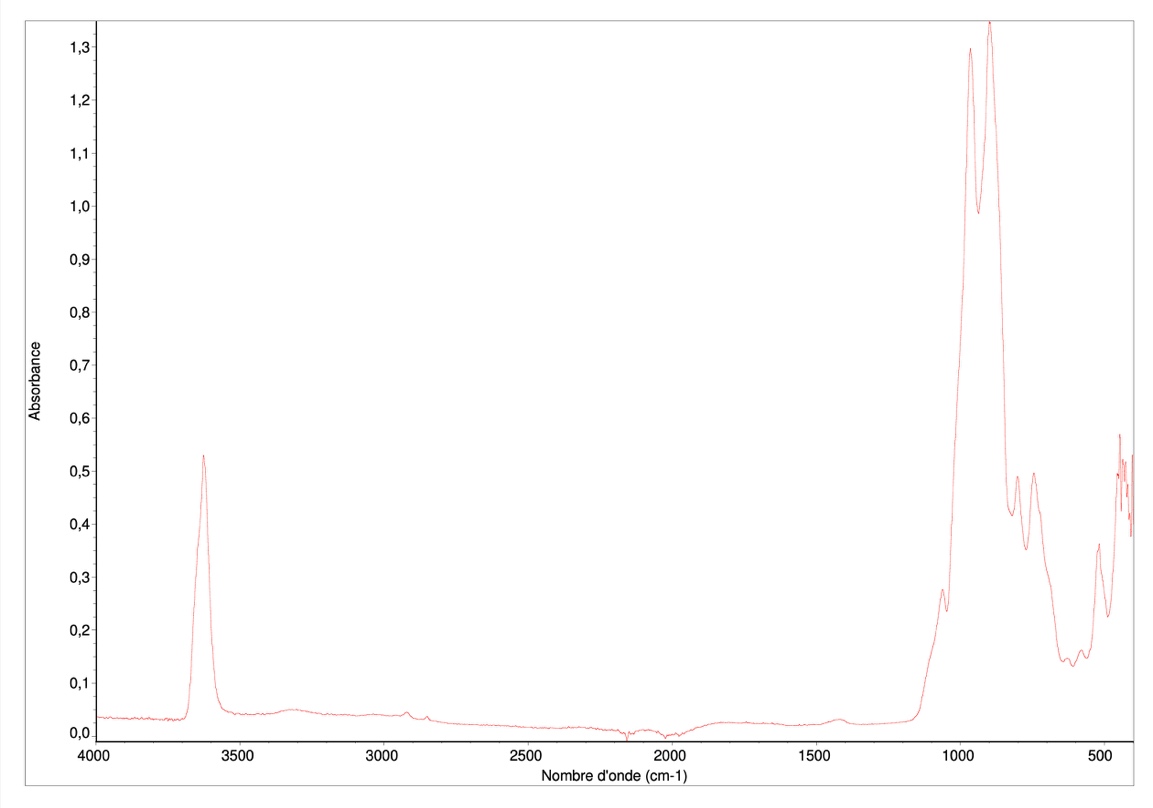


(a)

(b)

(c)

**Figure S4**. FT-IR spectrum of a) bear mica surface, b) spermidine-pretreated mica surface with deposited DNA network, c) mica pretreated with spermidine.


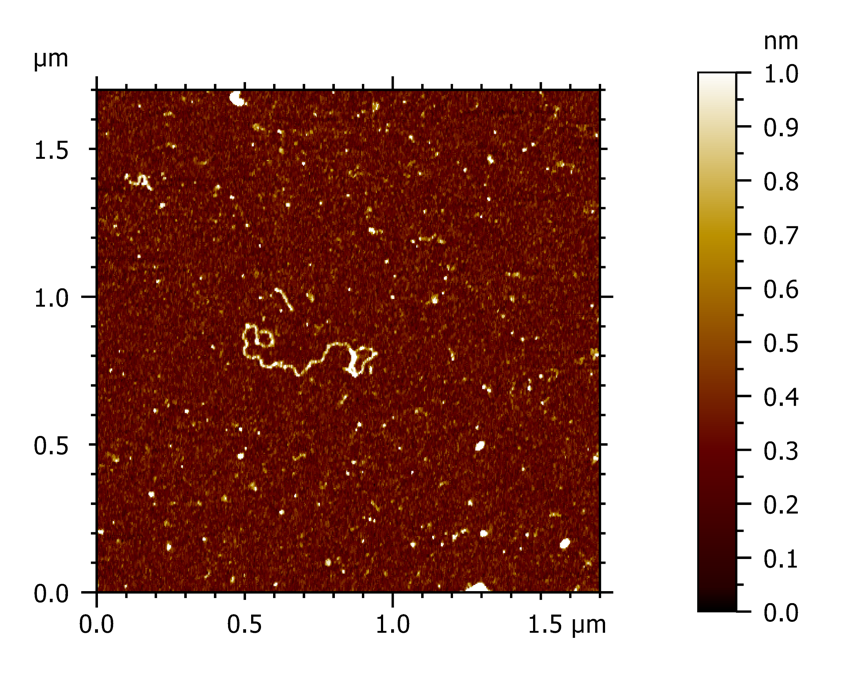


**Figure S.5** Single molecule DNA deposited on Ni-pretreated mica surface

**
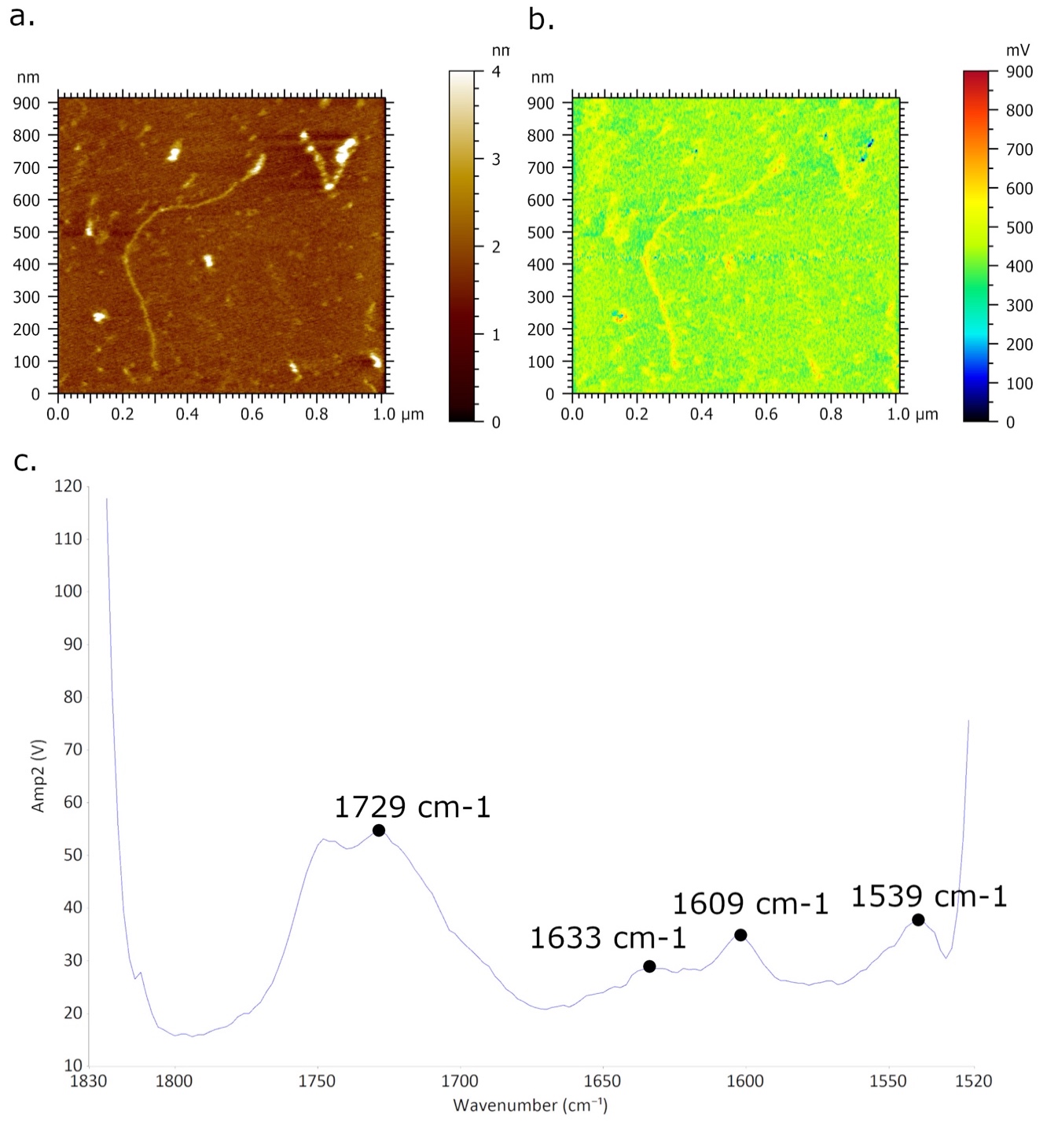
Figure S6** AFM-IR imaging of single DNA molecule deposited upon Ni^2+^ pretreated mica surface. Size of imaged area: 1.0 x 1.0 µm. a) AFM topography image of DNA and corresponding b) AFM-IR absorption map of single DNA recorded at optimized wavenumber 1728 cm^-1^ showing DNA of higher absorbance (bright yellow contour) regarding to mica surface as green background. c) AFM-IR spectra acquired and collected from twenty-six positions above single DNA contour showing the absorption maxima at 1729 cm^-1^ and 1748 cm^-1^ attributed to C=O carbonyl stretching of nucleobases.


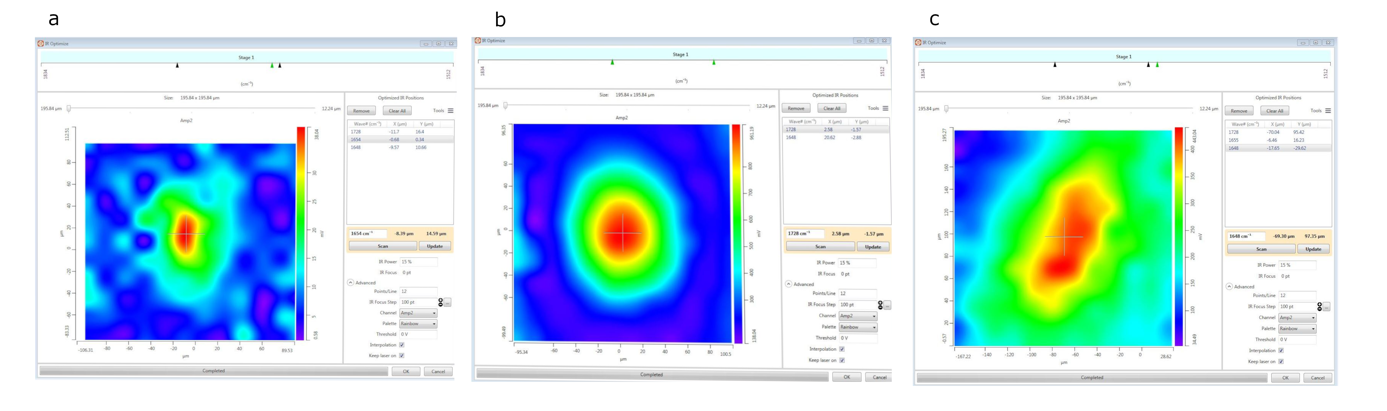


**Figure S7**: Examples of optimized focussed IR spot for a) 1654 cm^-1^, b) 1728 cm^-1^, and c) 1648 cm^-1^


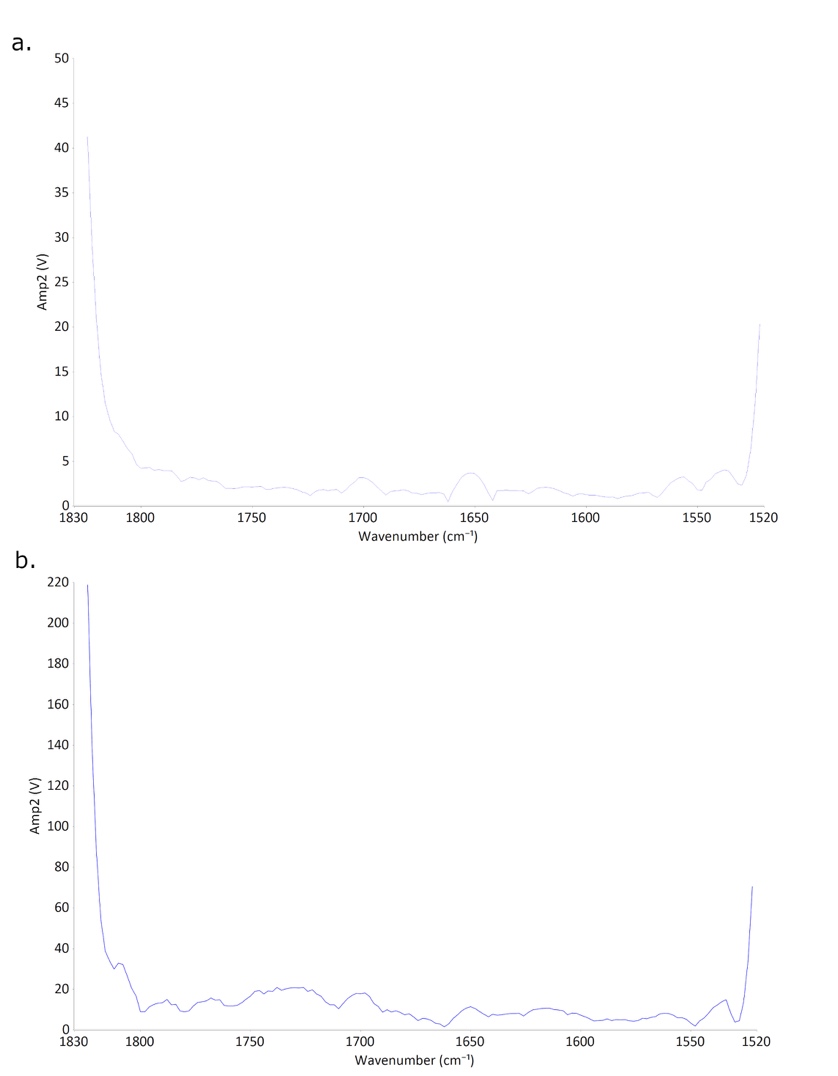


**Figure S8**: IR spectra of a) mica+ spermidine and b) mica + buffers


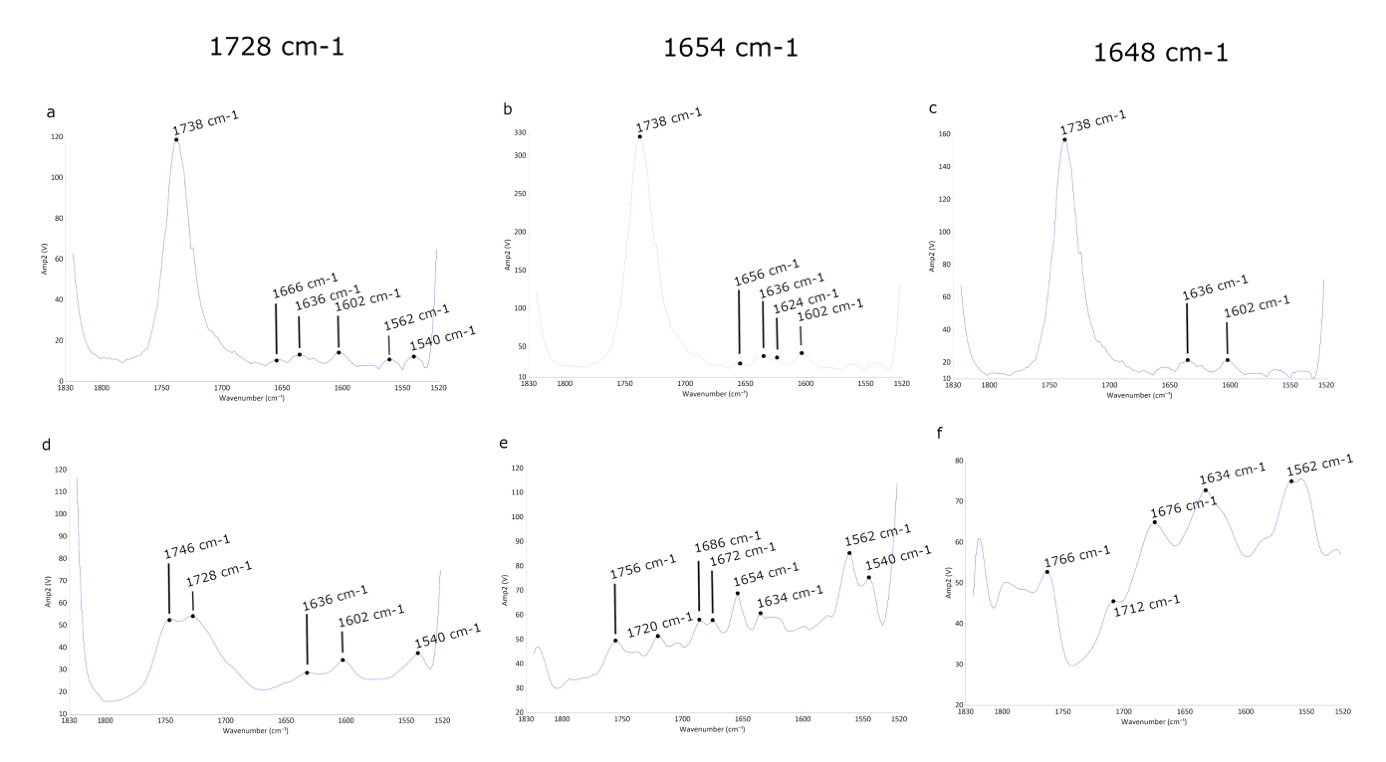


**Figure S9** IR-AFM spectrums acquired at optimized wavenumbers 1728 cm^-1^, 1654 cm^-1^ and 1648 cm^-1^ for DNA network (a-c) and single DNA molecule. Each spectrum were acquired with the laser power of 15%.
